# Regulation of T Cell Development and Activation by Creatine Kinase B

**DOI:** 10.1371/journal.pone.0005000

**Published:** 2009-04-01

**Authors:** Yafeng Zhang, Hai Li, Xiaoming Wang, Xiang Gao, Xiaolong Liu

**Affiliations:** 1 Laboratory of Molecular Cell Biology, Institute of Biochemistry and Cell Biology, Shanghai Institutes for Biological Sciences, Chinese Academy of Sciences, Shanghai, China; 2 Model Animal Research Center, Nanjing University, Nanjing, China; Oklahoma Medical Research Foundation, United States of America

## Abstract

Creatine kinase catalyzes the reversible transfer of the N-phosphoryl group from phosphocreatine to ADP to generate ATP and plays a key role in highly energy-demanding processes such as muscle contraction and flagellar motility; however, its role in signal transduction (which frequently involves ATP-consuming phosphorylation) and consequent cell-fate decisions remains largely unknown. Here we report that creatine kinase B was significantly up-regulated during the differentiation of double-positive thymocytes into single-positive thymocytes. Ectopic expression of creatine kinase B led to increased ATP level and enhanced phosphorylation of the TCR signaling proteins. Consequentially, transgenic expression of creatine kinase B promoted the expression of Nur77 and Bim proteins and the cell death of TCR signaled thymocyte. In addition, the activation, proliferation and cytokine secretion of T cells were also enhanced by the expression of creatine kinase B transgene. In contrast, treatment of T cells with specific creatine kinase inhibitor or creatine kinase B shRNA resulted in severely impaired T cell activation. Taken together, our results indicate that creatine kinase B plays an unexpected role in modulating TCR-mediated signaling and critically regulates thymocyte selection and T cell activation.

## Introduction

Intrathymic T cell development is critical for the establishment of a properly functioning adaptive immune system. T cell precursors generated in the bone marrow migrate to the thymus where their TCR genes are rearranged and their fates are dictated [1]–[3]. Thymocytes with defected TCR could not be signaled and go into a process of apoptosis termed “death by neglect”; Thymocytes expressing TCR with high affinity for self peptide- MHC molecules undergo negative selection and die locally in the thymus, thus being eliminated from the T cell pool [4]. Conversely, thymocytes that express TCR with low affinity for self peptide-MHC molecules receive survival signals, initiate positive selection of the cells and give rise to mature CD4 or CD8 T cells [5], [6]. Through positive and negative selection, an immunocompetent and self-tolerant T cell repertoire is generated [7], [8]. T cells that pass the selection leave the thymus and initiate immune surveillance in peripheral tissues where they may encounter their specific foreign antigen and become activated [9].

Stimulation of TCR by the peptide-MHC complex triggers a cascade of phosphorylation and dephosphorylation events in a spatially and temporally ordered manner in T cells [10]–[12], during which immune-receptor tyrosine-based activation motifs (ITAMs) of the CD3 molecules are phosphorylated by the Src-family tyrosine kinase Lck, phosphorylated ITAMs then recruit another tyrosine kinase Zap70 and facilitate the phosphorylation of Zap70 by Lck, and in turn activated Zap70 phosphorylates the adaptor proteins LAT and SLP-76 [11]. Phosphorylation of tyrosine residues on LAT and SLP-76 results in recruitment of a batch of other signaling proteins and subsequently leads to Ca^2+^ mobilization and activation of multiple pathways, including ERK, JNK, p38 and NF-κB pathways, which finally activate distinct nuclear factors involved in thymocyte differentiation, T cell proliferation and cytokine production [13]–[15].

The roles of various protein kinases in TCR signaling pathway have become relatively clear after extensive studies [10], [16]–[18]. Considering that all phosphorylation reactions catalyzed by these protein kinases require ATP as the phosphoryl donor [17], [19], it is likely that the intracellular ATP concentration and the ATP regeneration capacity of T cells have a strong impact on TCR signal strength. In fact, for different subsets of T-lineage cells at distinct developmental stages, their requirements of TCR signaling and competence to transduce TCR signal are quite different, which may be partially fulfilled by modulating their cellular ATP level. However, the generation, storage and usage aspects of ATP in T-lineage cells have been poorly investigated up to date.

The cellular ATP pool is relatively constant but ATP itself is rather unstable. It has been reported that creatine kinase (CK) helps keep the ATP pool constant through catalyzing the reversible transfer of the phosphoryl group from phosphocreatine (PCr) to adenosine 5′-diphosphate (ADP) [20]–[22]. CK genes are expressed in several tissues with highly fluctuating energy turnover, e.g. skeletal and cardiac muscle, brain and spermatozoa [23], [24]. Several isoenzymes of CK have been characterized: brain-type (Ckb), muscle-type (Ckm), and the mitochondrial CK isoenzymes (Ckmt1 and Ckmt2). It has been reported that dysregulated CK is associated with many diseases, such as heart disease, mental diseases, cancer and inflammatory diseases [25]–[29]. Despite that the properties and functions of CK in energy-demanding processes have been investigated extensively, its role in specific cellular signal transduction and consequential cell fate decisions remains largely unknown.

In attempt to obtain a better understanding of thymocyte development, we had used cDNA microarray technique to screen genes potentially involved in this process. Ckb was found to be stage-specifically expressed by this approach and was chosen for this study considering it directly participates in the regulation of ATP generation. Here we show that the expression levels of Ckb in double-positive (DP) thymocytes, single-positive (SP) thymocytes and T cells are marginal, high and median respectively, which is in accordance with their cellular ATP levels. We further show that ectopic expression of Ckb results in increased ATP level and enhanced TCR signaling, and transgenic expression of Ckb promotes premature thymocyte death and enhances T cell proliferation and cytokine production. And importantly, Suppression of Ckb activity by specific inhibitor or RNA interference attenuates TCR signaling and inhibits T cell activation. These data identify Ckb as an important regulator of T cell development and activation by tuning TCR signal strength.

## Results

### Ckb is stage-specifically expressed in T-lineage cells

**Figure 1:**
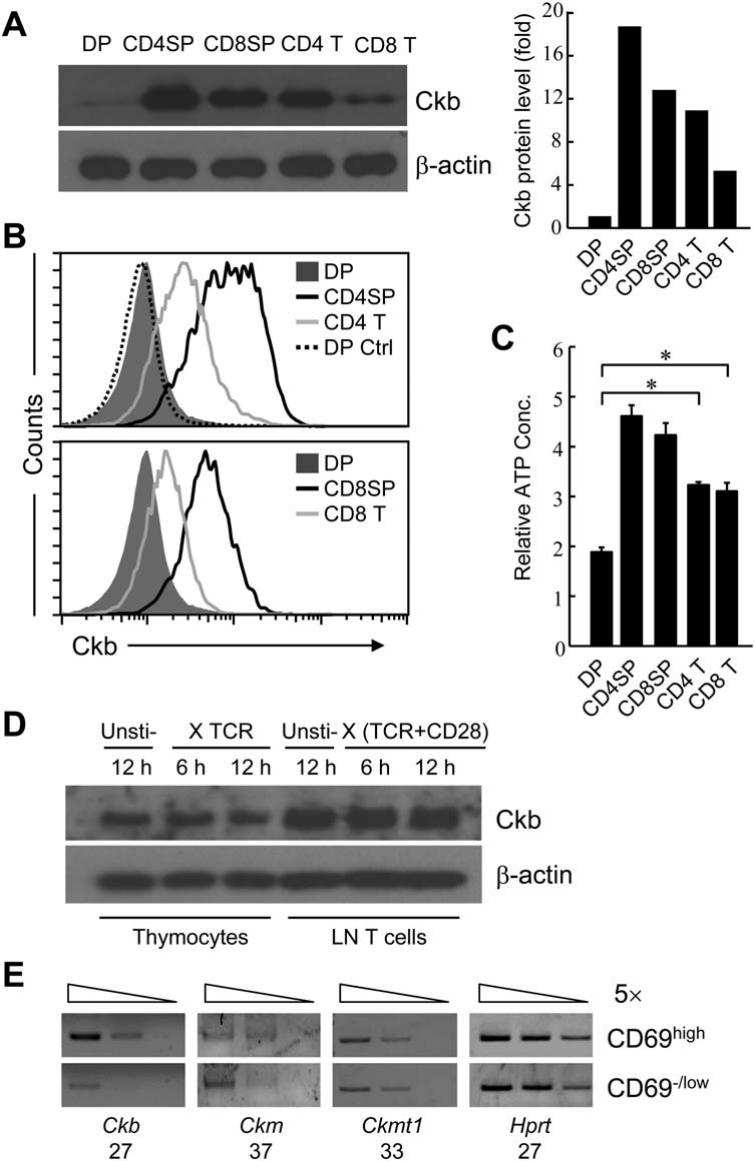
The expression profiles of Ckb in T-lineage cells. (A) The expression of Ckb in purified cells was analyzed by immunoblot with a rabbit polyclonal antibody against Ckb and the optical density of the bands was quantified and normalized against that of β-actin. (B) Flow cytometric analysis of Ckb expression in thymocytes and T cells. DP thymocytes stained with pre-serum was used for negative control (DP Ctrl). (C) The relative intracellular ATP concentration was measured in purified DP, CD4SP, CD8SP thymocytes and CD4, CD8 T cells (*, P<0.05). (D) Total thymocytes and lymphocytes were stimulated with anti-TCR (10 µg/ml) or anti-TCR (10 µg/ml) and anti-CD28 (10 µg/ml) respectively for 6 h or 12 h or unstimulated (Unsti-) and then analyzed for Ckb expression. (E) RT-PCR analysis showed that only Ckb is differentially expressed during thymocyte development. CD69^high^ and CD69^−/low^ thymocytes were sorted from C57BL/6 mice. Bands resolved on agarose gels show PCR products by 5-fold serial dilution of cDNA (wedges), numbers of PCR cycles used for amplification were written underneath the gene name. *ckmt2* was not detectable in these two subsets. All the results above are representative of three independent experiments.

Previous microarray analysis (GEO database, GSE2262) revealed that the transcription of Ckb was significantly higher in CD69^high^ thymocytes (comprising TCR signaled DP, CD4SP and CD8SP thymocytes) than in CD69^low^ thymocytes (unsignaled DP thymocytes only) [30]. Consistent with this observation, Western blot analysis showed about 18-fold increase in Ckb protein abundance from DP to CD4SP subsets, while surprisingly there was a about 1.5-fold decrease from CD4SP to CD4 T cell subsets (Figure 1A); similar expression pattern of Ckb was found in CD8-lineage cells. Intracellular staining was further used to depict the Ckb expression pattern in T-lineage cells. As shown in Figure 1B, DP thymocytes had the lowest Ckb expression, SP thymocytes had the highest expression level and expression in T cells was in the middle. Considering CK is a key enzyme that catalyzes ATP generation, the relative levels of ATP were next measured in the purified subsets. In accordance with the expression level of Ckb, the ATP concentration was lowest in DP thymocytes, highest in CD4SP and CD8SP thymocytes and middle in CD4 and CD8 T cells (Figure 1C).

Since Ckb expression was up-regulated along with the maturation of SP cells, we wondered if TCR signaling could directly promote Ckb expression. As shown in Figure 1D, the expression of Ckb was not enhanced in thymocytes and T cells after stimulation with anti-TCR or anti-TCR and anti-CD28 respectively; while CD69, as a positive control, was greatly up-regulated upon stimulation (Figure S1), suggesting that Ckb is not a direct target of TCR signaling. We also compared the expression levels of other CK isoenzymes between CD69^high^ and CD69^−/low^ thymocytes, RT-PCR analysis of transcriptions showed that only Ckb was differentially expressed (Figure 1E).

### Transgenic expression of Ckb promotes ATP generation and enhances TCR signal strength

**Figure 2:**
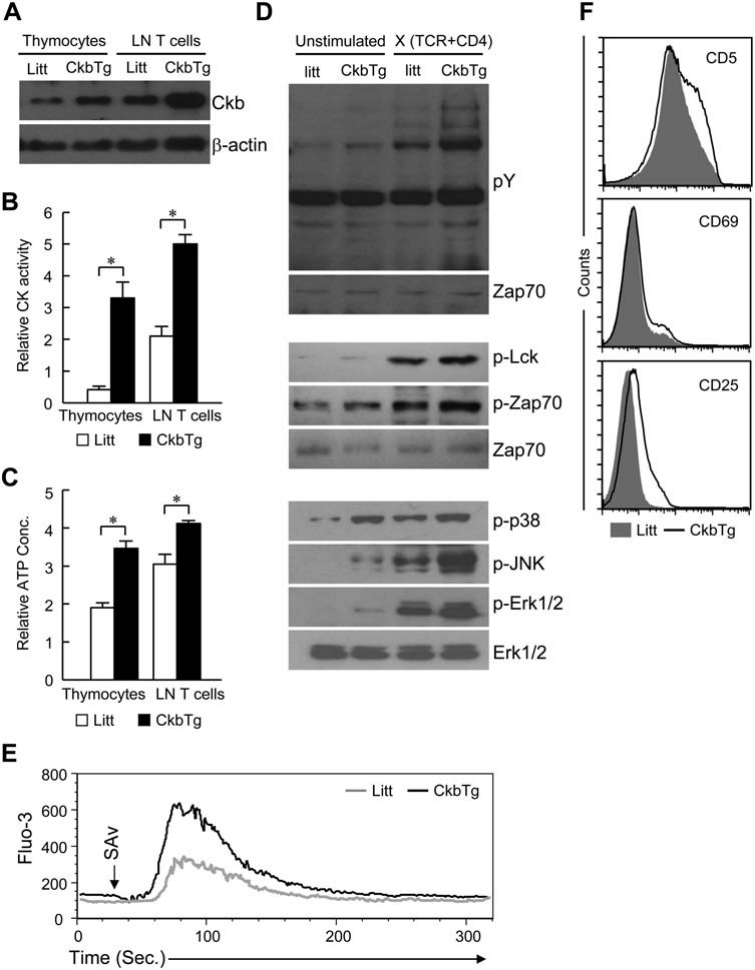
Transgenic Ckb promotes ATP generation and augments TCR signal strength. (A) Expression of Ckb protein was analyzed by immunoblot analysis of lysates from total thymocytes or lymphocytes, β-actin served as a loading control. (B) Creatine kinase activity in cell extracts was determined by using standard methods as described in Materials and Methods (*, P<0.05). (C) Detection of intracellular ATP concentration of beads-purified cells (*, P<0.05). (D) Analyses of phosphorylation of proteins involved in TCR signaling. FACS-purified DP thymocytes were left unstimulated or stimulated by anti-TCR plus anti-CD4 streptavidin-mediated crosslinking for 2 min, Shown on the top are tyrosine phosphorylation of total cellular proteins, membrane was stripped and reprobed for Zap70 as a loading control; Shown on the middle are tyrosine phosphorylation of Lck and Zap70, equal loading of the lanes was verified by anti-Zap70 immunoblotting; Phosphorylation of p38, JNK and Erk1/2 is shown on the bottom, membranes were stripped and reprobed for ERK1/2 as a loading control. (E) TCR-induced Ca^2+^ mobilization. Shown are the histograms of Ca^2+^ mobilization in control cells (gray line) and CkbTg (black line) DP thymocytes. Thymocytes were stimulated with biotin-conjugated anti-TCR and anti-CD4, cross-linked with streptavidin, and analyzed for Ca^2+^ mobilization. Arrows indicate the time points when straptavidin was added. (F) Flow cytometric analysis of the expression of CD5, CD69 and CD25 on DP thymocytes. The results are representative of three to four independent experiments. Litt, littermate; CkbTg, Ckb transgenic mouse.

Since Ckb is differentially expressed and critical for ATP metabolism, we speculated that it may participate in regulating thymocyte development and T cell function. Considering that Ckb is expressed at a low level at DP stage, overexpression model would provide a useful tool to investigate its effects on thymocyte selection. To this end, we generated Ckb transgenic (CkbTg) mice using a wild-type *Ckb* cDNA under the transcriptional control of human CD2-based regulatory elements, which conferred ectopic expression of Ckb in T-lineage cells. As shown in Figure 2A and Figure S2, the expression level of transgenic Ckb in DP thymocytes is similar to that of the endogenous Ckb in CD4SP thymocytes from littermate controls.

We then ascertained whether transgenic Ckb is functional, the total CK activity was measured and the results indicated that the CK activity was much higher in transgenic cells than in control counterparts (Figure 2B). Previous studies suggest that CKs mainly function to accelerate the rates of high energy phosphate exchange between PCr and ATP rather than increase intracellular ATP level [31]. However, we observed that the ATP level was increased by 1.6-fold in transgenic DP thymocytes and 1.4-fold in transgenic T cells compared with control cells respectively (Figure 2C). We then asked whether transgenic expression of Ckb affects TCR signaling, as shown in Figure 2D, the magnitude of tyrosine phosphorylation of either total cellular proteins (Top panel) or Lck and Zap70 (Middle panel) was measurablely enhanced, and the phosphorylation of p38, JNK and Erk1/2 was substantially increased in CkbTg DP thymocytes relative to their littermate counterparts in the presence of TCR stimulation (Bottom panel). Consistent with the above observations, TCR-induced Ca^2+^ mobilization was also enhanced in CkbTg DP thymocytes (Figure 2E). To further examine whether the CkbTg thymocytes were better signaled under the de facto situation, we measured the activation markers on the DP thymocyte surface. DP thymocytes from CkbTg mice significantly up-regulated the expression of CD5, CD69 and CD25 compared with respective counterparts from littermates (Figure 2F).

### Early expression of Ckb leads to loss of premature DP thymocytes

**Figure 3:**
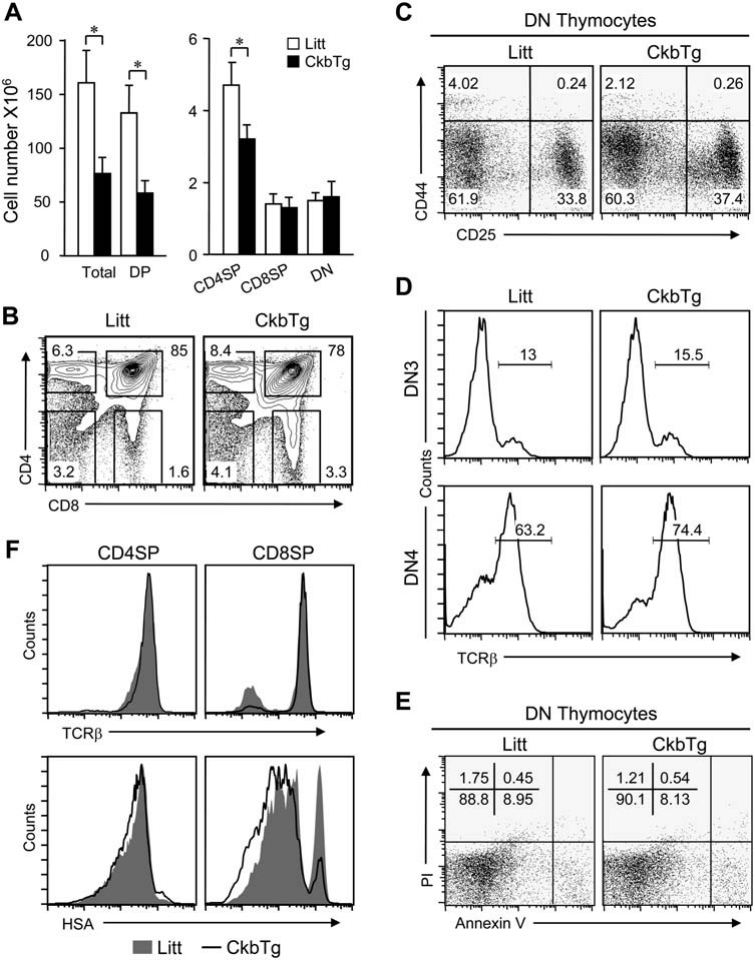
Flow cytometric analysis of thymocyte populations. (A) Comparison of thymocyte subsets in Ckb-transgenic mice and littermate controls. Bar graphs (error bars: SEM) indicate the average numbers of each thymocyte subset. The results were summarized from 4- to 5-week-old Ckb-transgenic mice (n = 5) and littermate control mice (n = 5) (*, P<0.05). (B) Dot plots indicate CD4 versus CD8 profiles of live thymocytes, numbers next to boxes indicate the percentage of cells in that box. (C) The relative distribution of DN subsets revealed by CD44 and CD25 staining. (D) Intracellular staining for TCRβ expression in DN3 and DN4 thymocytes, Numbers on the graphs represent the percentage of TCRβ positive cells. (E) Freshly isolated thymocytes were stained with anti-CD4 and anti-CD8, followed by dual-labeling for annexin V and PI and analyzed by flow cytometry, the percentages of cells within each quadrant are shown in the upper left quadrants. (F) Detection of TCR and HSA expression on CD4SP and CD8SP thymocytes from CkbTg mice and littermate controls. The results are representative of three to fiver independent experiments. Litt, littermate; CkbTg, Ckb transgenic mouse.

To examine if the transgenic expression of Ckb altered thymocyte development, cell numbers were determined and the results showed that the total thymic cellularity of CkbTg mice was reduced approximately by half (Figure 3A, left). We next analyzed thymocyte populations by staining of CD4 and CD8 surface markers, compared with nontransgenic littermates, CkbTg mice showed a decrease in the proportion of DP and CD4SP thymocytes and an increase in the proportions of the other subsets (Figure 3B). Statistically, the observed drop in cellularity mainly came from DP subset, which reduced about two thirds in transgenic mice compared to littermates (Figure 3A, right). The decrease in DP cellularity in CkbTg thymus may be due to defects in early DN development, analysis of the distribution, TCRβ expression and viability of DN subsets showed that these events were comparable between CkbTg thymocytes and their littermate counterparts (Figure 3, C, D and E), indicating that TCR β selection is normal in CkbTg mice. To evaluate the maturity of CD4 and CD8 SP thymocytes in CkbTg mice, the expression of TCR and heat-stable antigen (HSA) was analyzed and no significant difference was detected between CkbTg and littermate SP thymocytes (Figure 3F).

### Enforced expression of Ckb enhances the apoptosis of TCR signaled thymocytes

**Figure 4:**
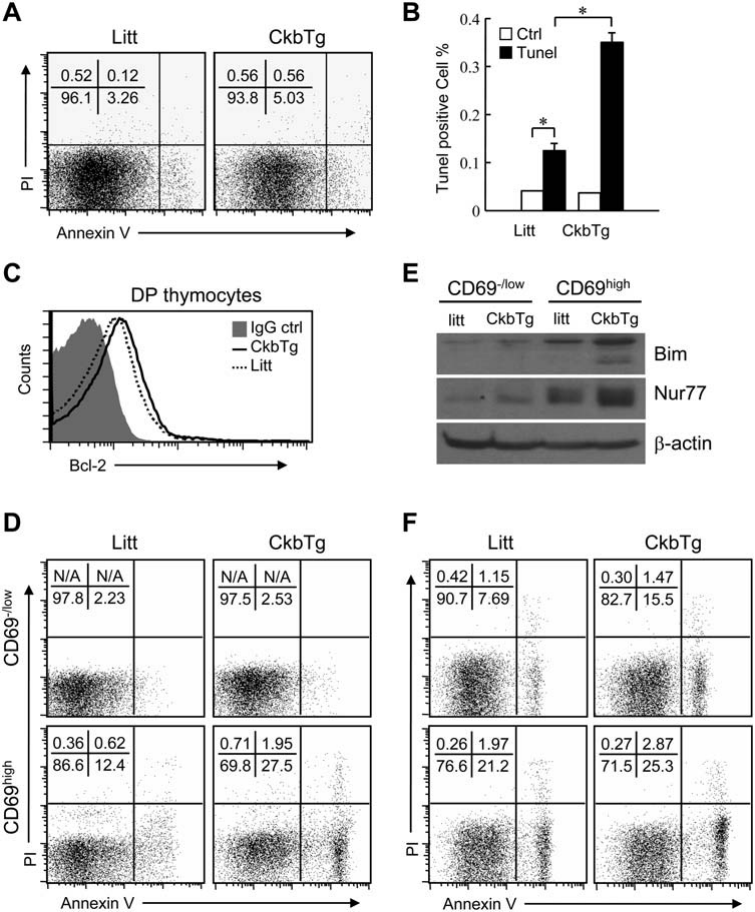
Transgenic expression of Ckb promotes premature death of DP thymocyte. (A) Apoptotic and dead DP thymocytes were discriminated on the basis of a double-labeling for annexin V and PI and analyzed by flow cytometry, the percentages of cells within each quadrant are shown in the upper left quadrants. (B) Apoptotic DP thymocytes were measured by TUNEL assay (Ctrl, TUNEL reaction mixture without TdT enzyme solution; *, P<0.05). Thymocytes used in both (A) and (B) were freshly prepared. (C) Flow cytometric analysis of Bcl-2 expression in DP thymocytes. (D) Flow cytometric analysis of apoptotic CD69^−/low^ and CD69^high^ DP thymocytes from CkbTg mice and their littermate controls using Annexin V and PI staining. The percentages of cells within each quadrant are shown in the upper left quadrants, N/A, not applicable. (E) Immunoblot analysis of Bim and Nur77 expression in FACS-sorted CD69^−/low^ and CD69^high^ DP thymocytes, β-actin served as a loading control. (F) Thymocytes were stimulated with different concentrations of plate-bound anti-TCR and anti-CD28 antibodies for 12 h and then used for apoptotic analysis (Top, anti-TCR (H57-597) 1 µg/ml, anti-CD28 (37.51) 1 µg/ml. Bottom, anti-TCR (H57-597) 10 µg/ml, anti-CD28 (37.51) 10 µg/ml), the percentages of cells within each quadrant are shown in the upper left quadrants. Data are representative of three independent experiments. Litt, littermate; CkbTg, Ckb transgenic mouse.

According to the above results, the loss of DP thymocyte in CkbTg mice is likely due to increased cell death. Indeed, we found that CkbTg mice have more DP thymocytes undergoing apoptosis in vivo as detected by annexin V and propidium iodide (PI) staining, an approximately 1.5-fold increase in early apoptotic (Annexin V^+^PI^−^) DP thymocytes was detected in CkbTg mice compared with that of their littermate controls (Figure 4A). The enhanced apoptotic thymocytes in CkbTg mice were further confirmed by TUNEL assay (Figure 4B). Since CkbTg DP thymocytes were hyperactivated (Figure 2, D–F), we assumed that enhanced apoptosis of DP thymocytes in CkbTg mice was caused by activated cell death rather than death by neglect. To test this possibility, we first assessed CkbTg thymocytes for the expression of antiapoptotic Bcl-2 molecule, which was normally up-regulated in signaled DP thymocytes to protect thymocytes from death by neglect [32], as shown in Figure 4C, CkbTg DP thymocytes expressed the similar amount of Bcl-2 as control thymocytes; we then compared the difference in apoptosis of TCR signaled (CD69^high^) and unsignaled (CD69^−/low^) DP thymocytes from CkbTg and control mice respectively and found that the difference was greater in the comparison of CD69^high^ cells than in that of CD69^−/low^ cells (Figure 4D), which indicated that specific deletion was occurred after TCR triggering. In addition, we analyzed the expression of orphan nuclear receptor Nur77 and the proapoptotic protein Bim, which are crucial mediators of negative selection [33], [34], by using the same subsets as used in the above experiment. Interestingly, Immunoblot revealed that the expression of Nur77 and Bim was up-regulated in CkbTg thymocytes and greater difference was found between CD69^high^ DP thymocytes than between CD69^−/low^ DP thymocytes (Figure 4E), indicating that more TCR signaled thymocytes were undergoing negative selection in transgenic mice.

We then asked whether the enhanced negative selection of CkbTg thymocytes is mediated by the altered TCR signal strength, to address this issue, we stimulated thymocytes with plate-bound anti-TCR and anti-CD28 to mimic negative selection in vitro [35]. As shown in Figure 4F, apoptosis of stimulated DP thymocytes was clearly detectable by Annexin V versus PI staining, however, more apoptotic cells were detected in CkbTg DP thymocytes than in control DP thymocytes from two separate experiments, suggesting that the enhanced negative selection induced by transgenic expression of Ckb is TCR signaling-dependent.

**Figure 5:**
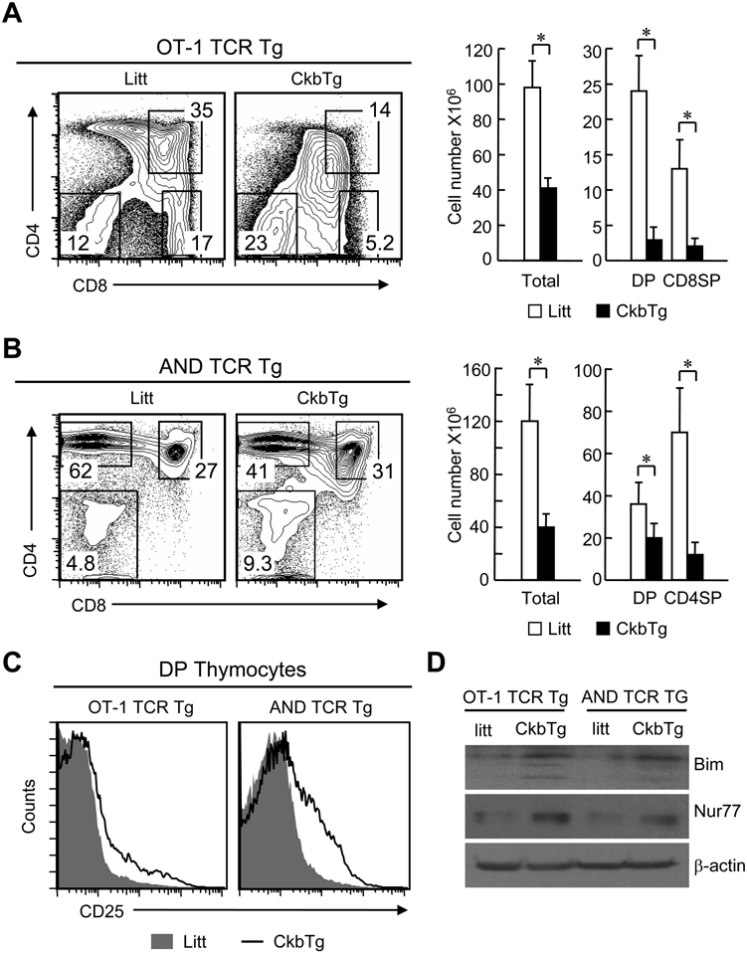
Ectopic expression of Ckb impairs thymic positive selection. (A, B) Dot plots indicate CD4 versus CD8 profiles of total thymocytes from TCR transgenic or Ckb and TCR double transgenic mice, numbers next to boxes indicate the percentage of cells in that box. The cell numbers of total thymocytes or different subsets are shown on the right. (A), MHC Class I restricted OT-1 TCR transgenic; (B) MHC Class II restricted AND TCR transgenic (*, P<0.05). (C) Detection of surface CD25 expression on DP thymocytes. (D) Immunoblot analysis of Bim and Nur77 expression in total thymocytes, β-actin served as a loading control. Litt, littermate; CkbTg, Ckb transgenic mouse.

To examine the influence of transgenic expression of Ckb on positive selection in the thymus, OT-1(MHC Class I-restricted) and AND (MHC class II-restricted) TCR transgenic mice were separately crossed to the CkbTg mice. Normally, the OT-1 or AND TCR transgenic thymocytes express the same MHC restricted TCR and undergo positive selection of CD8 and CD4 T cells respectively. Intriguingly, on Ckb transgenic background, positive selection of TCR transgenic thymocytes was greatly impaired as evidenced by the dramatically decreased number of both DP and SP thymocytes (Figure 5, A and B). Notably, the Ckb and TCR double transgenic DP thymocytes expressed CD5 and CD69 at slightly lower levels (Figure S3), which might reflect quick death of CD5^high^ and CD69^high^ cells, they had higher CD25 expression compared with their counterparts from single TCR transgenic mice (Figure 5C). And more, the expression of Nur77 and Bim was up-regulated in the Ckb and TCR double transgenic thymocytes (Figure 5D). These data reveal that transgenic expression of Ckb promoted TCR-mediated apoptosis and thus contributed to increased premature thymocyte death in CkbTg thymus.

### Ectopic Ckb expression augments T cell activation

**Figure 6:**
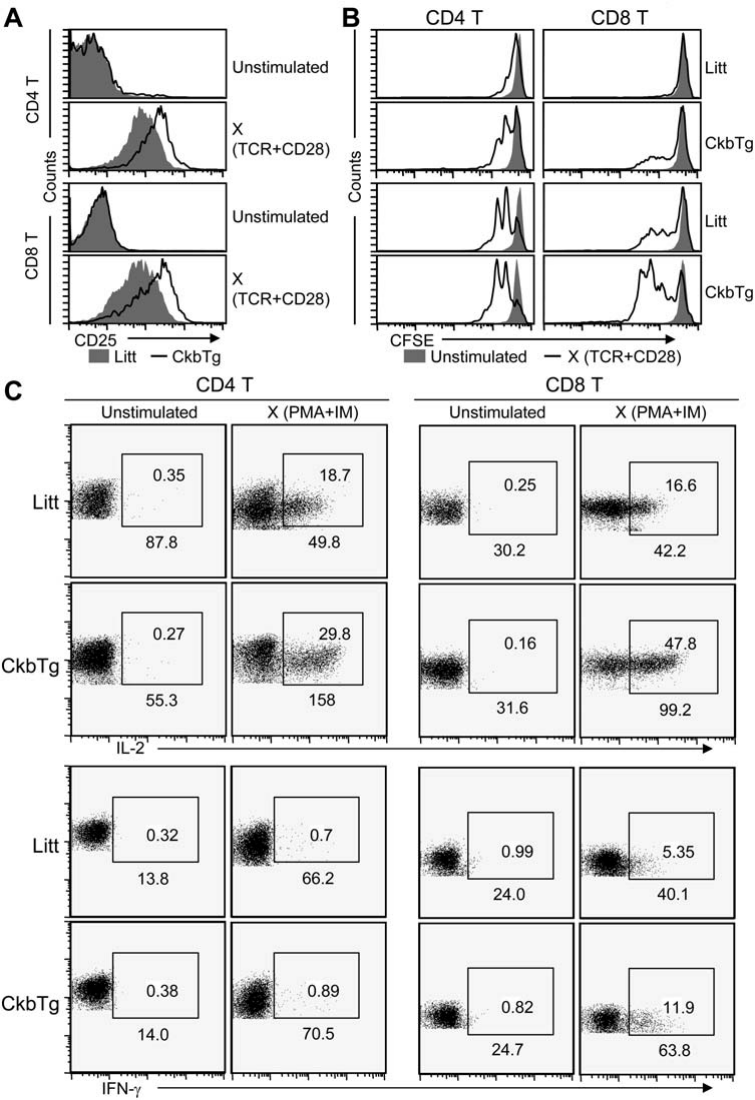
Ecotopic expression of Ckb enhances TCR sensitivity and T cell response. (A) Flow cytometric analysis of the activation marker CD25 on CD4 and CD8 T cells, cells were stimulated with plate-bound anti-TCR (10 µg/ml) and anti-CD28 (10 µg/ml) antibodies or left unstimulated for 16 h. (B) CFSE-labeled lymphocytes were stimulated with plate-bound anti-TCR (top 0.5 µg/ml, bottom 2 µg/ml) and anti-CD28 antibodies (top 0.5 µg/ml, bottom 2 µg/ml) or left unstimulated. Cells aliquots were analyzed for CFSE fluorescence as an indicator of cell division. Analyses were gated on CD4^+^ or CD8^+^ cells as indicated. (C) Analysis of cytokine expression. LN T cells were isolated from 6-week-old CkbTg and Littermate mice, left unstimulated or stimulated with PMA and IM for 4 h and then analyzed for CD4, CD8, IL-2 and IFN-γ expression. Values in the gating boxes indicate the percentage of IL-2^+^ or IFN-γ^+^ cells and numbers underneath the gating boxes indicate the MFI of IL-2 and IFN-γ. The results are representative of three independent experiments. Litt, littermate; CkbTg, Ckb transgenic mouse.

In contrast to the population variation found in the thymus, the relative proportions and absolute numbers of peripheral CD4 and CD8 T cell were similar between transgenic and littermate mice (Figure S4). Given the positive impact of Ckb on TCR signal transduction, we next examined whether Ckb participates in the regulation of T cell activation. As shown in Figure 6 A and B, compared with their littermate counterparts, CkbTg T cells expressed high levels of CD25 and proliferated strongly after stimulation with immobilized anti-TCR and anti-CD28 antibodies. Furthermore, the induction of IL-2 and IFN-γ was analyzed by intracellular staining after stimulation with PMA and IM as described [36], as shown in Figure 6C, both the mean fluorescence intensity (MFI) and the frequency of IL-2 and IFN-γ were increased in CkbTg T cells. Taken together, the above results indicate that Ckb protein significantly improves TCR signaling capacity and suggest that Ckb plays an important role in T cell activation.

### Down-regulation of Ckb reduces T cell sensitivity to TCR stimulation

**Figure 7:**
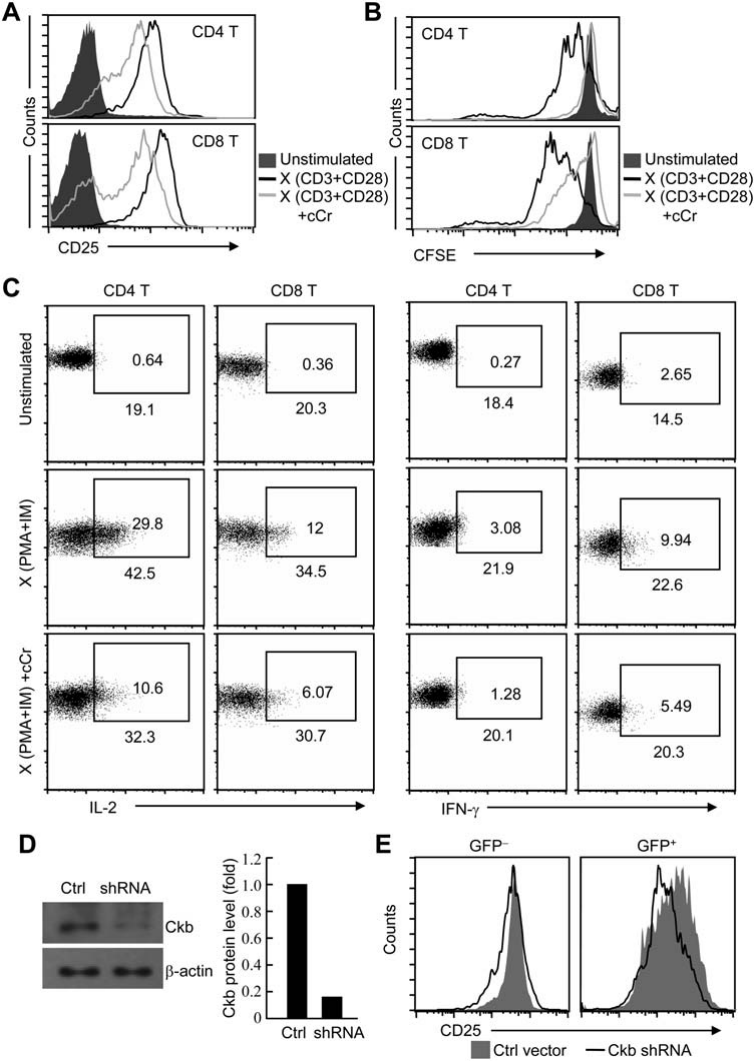
Suppression of Ckb activity attenuates TCR-mediated responses in T cells. (A) Flow cytometric analysis of CD25 expression on CD4 and CD8 T cells, cells were stimulated with plate-bound anti-CD3 and anti-CD28 antibodies (10 µg/ml each) with or without cCr or left unstimulated for 12 h, cCr were used at 50 mM. (B) Proliferation of CFSE-labeled lymphocytes was detected as described in Figure 6B. To be note, the concentration of cCr was 25 mM and the concentration of anti-CD3 and anti-CD28 is 5 µg/ml each. (C) Analysis of cytokine production, cell were treat with or without cCr (50 mM) for 12 h, then left unstimulated or stimulated with PMA and IM for 4 h and analyzed for CD4, CD8, IL-2 and IFN-γ expression. Values in the gating boxes indicate the percentage of IL-2^+^ or IFN-γ^+^ cells and numbers underneath the gating boxes indicate the MFI of IL-2 and IFN-γ. (D) Down-regulation of Ckb expression by lentiviral-mediated RNA interference. T cells were infected with either control (Ctrl) or Ckb-specific shRNA (shRNA), cells were then sorted on the basis of GFP expression and GFP positive (effectively infected) cells were subjected to Immunoblot analysis with Ckb antibodies, the optical density of the bands was quantified and normalized against that of β-actin. (E) Flow cytometric analysis of CD25 expression on T cells. T cells were infected with either control or Ckb-specific shRNA and then stimulated with plate-bound anti-TCR and anti-CD28 antibodies (10 µg/ml each). Analyses were gated on GFP^−^ (uninfected) or GFP^+^ (infected) cells. The results are representative of two to three independent experiments.

Finally, we examined whether Ckb is necessary for T cells to response properly. To achieve this, T cells were stimulated with anti-TCR and anti-CD28 antibodies in the absence or presence of cyclocreatine (cCr), one of the most kinetically active analog of creatine. Both cCr and creatine are substrates for CK and have been shown to modulate rates of ATP production, cCr was widely used as specific inhibitor to block the enzymatic activity of CK [37]–[40]. Cell viability was not affected in the presence of cCr (data no shown); however, the CD25 expression and proliferation of T cells were greatly impaired (Figure 7, A and B). In addition, by using the same approach described in Figure 6C, we found that the synthesis of IL-2 and IFN-γ was down-modulated in cCr treated T cells compared with those without treatment after PMA and IM stimulation (Figure 7C). RNA interference (RNAi) technology was further used to address the effect of depletion of endogenous Ckb on T cell activation. As shown in Figure 7D, the functional Ckb shRNA oligonucleotides effectively knocked down the expression of Ckb, accordingly, the response of transfected T cells to TCR stimulation was substantially attenuated as indicated by CD25 expression (Figure 7E). Although whether the impaired T cell activation was resulted from altered TCR signaling alone or in combination with other pathways is unclear here because ATP is pivotal for the phosphorylation reactions in general, we conclude that intact Ckb activity is important for full T cell activation from the above observations.

## Discussion

It is well known that CKs play key roles in energy metabolism in high energy-consuming tissues like brain and skeletal muscle through providing a temporal and spatial ATP buffer to maintain cellular energy homeostasis [20], [21]. While cellular ATP is used not only for fitting the fluctuating energy demands, but also for many other biological events, such as protein phosphorylation, cAMP generation and so on. Tissue specific and developmentally regulated CKs may integrate metabolic and apoptotic signals to mount other cellular response besides muscle contraction and neuron function [24], [38], [41], [42]. In this study, we have demonstrated an unexpected role of Ckb in regulating thymocyte development and T cell activation via modulating TCR signal strength.

Consistent with the observation that cytosolic isoenzymes of CK, as a catalyzer, mainly promote ATP generation rather than ATP consumption [43], we found CkbTg thymocytes and T cells had more ATP than their control counterparts (Figure 2C). By using cell-based assays, we showed that CkbTg DP thymocytes were TCR-signaled greater than their littermate counterparts (Figure 2, D–F), which might be not only induced by the increased ATP level but also the enhanced ability to buffer the ATP pool due to the ectopic expression of Ckb. Moreover, CkbTg T cells were found to be hypersensitive to TCR stimulation as evidenced by enhanced cytokine production and proliferation (Figure 6), on the other hand, T cell activation was greatly down-regulated when Ckb expression was silenced or its enzymic activity was blocked (Figure 7). Therefore, our results indicated that Ckb plays an important role in determining signal strength from the TCR.

The differentiation of DP thymocytes into CD4 or CD8 SP cells in the thymus is necessary for the establishment of immunocompetence of the cells, which is accomplished by reprogramming of a complex array of gene expression to fulfill diverse biological functions, including up-regulation of the antiapoptotic Bcl-2 for survival and of the IL-2Rα chain for cell proliferation [12], [44]. Previous reports showed that glucose transporter molecules are up-regulated during the thymocyte maturation, which improve nutrient uptake and increase metabolic activity and thus to provide the metabolic energy needed to activate new genetic programs [45], [46]. Here we report that Ckb, a novel metabolic regulator of T lineage cells, plays a critical role in modulating immune responses such as cell proliferation and cytokine secretion. Considering that TCR signals induce a serial of protein phosphorylation reactions, Ckb could work as an “amplifier” to enhance TCR signaling in multiple points through rapid providing cellular ATP, which finally leads to turn-on of specific factors for immune function in response to pathogen infection. Therefore, the up-regulation of Ckb expression from premature DP thymocytes to functionally mature SP and T cells may well be important for T cells to elevate their sensitivity to the limited foreign antigen.

We have noted that Ckb is more highly expressed by CD4SP thymocytes than CD8SP thymocytes, whether Ckb participates in regulating CD4/CD8 lineage decision is not directly addressed here, however, the CD4SP/CD8SP ratio in CkbTg mice differed from that in littermate mice (Figure 3B), which indicates altered lineage commitment. Intriguingly, we found that early expression of Ckb strongly promotes activated thymocyte death due to the enhanced TCR signaling, suggesting that Ckb is involved in this process. Another interesting feature is that Ckb is expressed at highest level in SP thymocytes and at relatively low level in T cells (Figure 1, A and B), this unique expression pattern may well facilitate the establishment of central T cell tolerance. The process of negative selection of thymocytes initiates as early as DP stage and will prolong to SP stage [8], [47]. At SP stage, the TCR signal strength is greatly enhanced due to the highest Ckb expression (rapid ATP regeneration and relative high ATP level), thus, the auto-reactive thymocytes are terminally proofread at this checkpoint and ultimately lead to activated cell death; and importantly, T cells that pass selection will down-regulate Ckb, and thereafter avoid being hyperreactive to self antigen in the periphery.

In this study, we have focused on the impact of Ckb on TCR signal strength, we demonstrated that Ckb tunes the threshold of TCR signaling leading to thymic selection and T cell proliferation; whether and how Ckb regulates other signaling pathways such as cytokine and chemokine pathways were not investigated. Ckb may also have similar impact on these signals, and future experiments will strive to address these issues.

## Materials and Methods

### Mice

C57BL/6, OT-1 and AND TCR transgenic mice were obtained from The Jackson Laboratory. CkbTg mice were generated by over-expression of Ckb under control of the human CD2 promoter and locus control region in the transgenic expression vector p29△2 (gift of Paul Love), two transgenic founders with similar phenotype were generated and one line was used in this study. Mice were maintained in SPF facility and analyzed between 5 and 10 weeks of age. All animal experiments were approved by the institutional animal use committee of the Shanghai Institutes for Biological Sciences, Chinese Academy of Sciences.

### Antibodies and reagents

The following monoclonal antibodies were from BD-Pharmingen: anti-TCRβ (H57-597), anti-CD4 (RM4.4 and GK1.5), anti-CD5 (53-7.3), anti-CD8α (53-6.7), anti-CD28 (37.51), anti-CD69 (H1.2F3), anti-TCR Vβ5 (B20.1), anti-Vα11 (RR8-1), anti-CD25 (2A3), anti-HSA (M1/69), anti-Bcl-2 (100) and anti-Nur77 (12.14). Anti-Erk1/2 (sc-154), anti-Bim (sc-11425), anti-phospho-Erk1/2 (sc-16981-R) and anti-phospho-P38 (sc-17852-R) were from Santa Cruz Biotechnologies. Anti-phospho-JNK(Thr183/Tyr185) was from Cell Signaling Technology, Inc. Polyclonal rabbit anti- mouse Ckb antibody was generated by our lab. In brief, Ckb fragment (encoding 1–143 amino acids of mouse Ckb) was constructed into pGEX-4T-1 (Amersham Pharmacia Biotech) vector and GST-fusion protein was expressed according to the manufacturers' protocols. The purified GST fusion protein was used as the antigen to immunize rabbits for generation of anti-Ckb polyclonal antibodies according to standard protocol. cyclocreatine (cCr) was purchased from Sigma-Aldrich.

### Cell preparation, purification and staining

Thymocyte and lymphocyte suspensions were prepared and were stained for the expression of cell surface markers as described [48]. A two-laser FACSCalibur (BD Biosciences) with four-decade logarithmic amplification was used for flow cytometry analysis, typically on 3×10^5^ cells/sample. Cells were stained for intracellular Ckb or Bcl-2 after 10 min fixation (2% formaldehyde PBS, room temperature) and 5 min permeabilization in IC staining buffer (0.03% saponin, 0.1% BSA, 0.1% NaN3 in PBS). For intracellular Bcl-2 staining, the cells were incubated sequentially with PE-conjugated anti-mouse Bcl-2, followed by staining for cell surface expression of CD4 and CD8. For the intracellular Ckb staining, the cells were firstly incubated with anti-Ckb, and then PE-conjugated anti-rabbit IgG, followed by cell surface staining. Results were analyzed using FlowJo software (Treestar). Live cells were identified by forward and side scatter or by propidium iodide (PI) gating.

### T cell proliferation, activation and apoptosis

T cell proliferation and stimulation were done as described [30]. T cells were labeled with CFSE (Molecular Probes, Eugene OR) at 1 µM in HBSS for 10 min at 37°C, washed with RPMI containing 10% FCS, and were incubated on plates coated with H57-597 and GK37.51 antibodies. For measurement of cell apoptosis, freshly isolated thymocytes were three-color stained with FITC-annexin V (Bender MedSystems), PE- conjugated anti-CD8 antibody, and APC- conjugated anti-CD4 antibody. Apoptotic cells were detected with annexin V and PI staining of the cells. For the activation of T cells, PMA and IM were used at concentrations of 10 ng/ml and 10 µg/ml respectively.

### Determination of intracellular ATP level

DP, CD4SP thymocytes and CD4 T cells were isolated using anti-CD4 (GK1.5), anti-CD8 (53-6.7) magnetic beads (Miltenyi Biotec) as described [30]. Cellular ATP level was determined by luciferin-luciferase-based assay. Aliquots containing 0.5×10^6^ purified thymocyte subsets or total thymocytes were processed using the ATP Bioluminescence Assay kit (from Beyotime) according to standard protocol.

### Reverse transcription polymerase chain reaction (RT-PCR)

RT-PCR analyses were done as described [48]. PCR products were separated by agarose gel electrophoresis and were visualized by ethidium bromide staining. PCR primers used were as follows: Ckb sense 5′-ATCCATCGCCCCTGCTTCGT-3′ and anti-sense 5′-GCTCATCACTGGGCTGGTAG-3′; Ckm sense 5′-GTCACCACCACCTCCACAGC-3′ and anti-sense 5′-ATGGCGGTCCTGGATGATGG-3′; Ckmt1 sense 5′-TGGGATACATTTTGACTTGCC-3′ and anti-sense 5′-TCATACCAGCAGACAGTTTACC-3′; Hprt sense 5′-CCTGCTGGATTACATTAAAGCACTG-3′ and anti-sense 5′-TTCAACACTTCGAGAGGTCCT-3′.

### Lentivirus-mediated RNA interference

HIV lentiviral vector pLL3.7 for shRNA expression is prepared as described previously [49]. The Ckb-specific-shRNA was constructed and transfected into packaging cells to produce retroviral supernatants (detailed procedures available on request). 1×10^6^ spleen cells were resuspended in 1 ml of retroviral supernatant containing 20 µg polybrene (Chemicon International), centrifuged at 1,000 g for 90 min at 25°C, and placed into fresh media for 2 d. Cells were then stimulated with plate-bound anti-TCR (10 µg/ml) and anti-CD28 (10 µg/ml) antibodies and followed by flow cytometry analysis, the pLL3.7 vector was used as control. The sense strand sequence of the Ckb shRNA is 5′-ACTACTCATTGAGATGGAG-3′

### Calcium flux assay

Thymocytes were preloaded with Fluo-3 AM at 5 µg/ml for 30 min at 37 °C, then surface stained with anti-CD8-APC as well as biotinylated anti-TCR and anti-CD4 (1 mg/ml), finally washed and resuspended (2×10^6^/ml) in HBSS and 2% FCS. After a baseline was established at quiescence, Ca2^+^ flux was induced by the addition streptavidin (4 µg/ml) for cross-linking. The resultant flux in Ca2+ was measured for 5 min by flow cytometry.

### Intracellular cytokine production analysis

Flow-cytometric analysis of cytokine production was performed as described [36]. Lymph node cells were stimulated in vitro with PMA (10 ng/ml) and IM (10 µg/ml) for 4 h in the presence of BFA (BD PharMingen). After culture, stimulated and unstimulated control cells were fixed for 10 min in room temperature in 2% formaldehyde. Then, cells were stained for surface expression of CD4, CD8, and intracellular cytokine staining antibodies according to the manufacturer's (BD Biosciences) protocol.

### TUNEL assay

TUNEL assay was performed using a kit (in situ cell death detection kit with fluorescein; Roche Molecular Biochemicals) according to the protocol provided by the manufacturer [50]. Briefly, 1×10^6^ thymocytes were fixed for 10 min (2% formaldehyde PBS, room temperature), and incubated with anti-CD4 and anti-CD8 for cell surface staining. After permeabilization with IC staining buffer, thymocytes were further incubated for 1 h at 37°C with 50 µl of the TUNEL solution or control solution. The apoptotic thymocytes were analyzed by flow cytometry.

### Creatine kinase activity assay

We performed creatine kinase activity assay with a kit according to the manufacturer's protocol (Beyotime). 20×10^6^ thymocytes were lysed in lysis buffer, and then the creatine kinase activity was determined by the rate of formation of NADPH from creatine phosphate using standardised methods [39].

### Statistical analysis

Differences between experimental and control results (mean±SE median) were analyzed by t test. Probability values of P<0.05 were considered significant.

## Supporting Information

Figure S1 Flow cytometric detection of CD69 expression following TCR stimulation. Total thymocytes and lymphocytes were stimulated with anti-TCR (10 µ/ml) or anti-TCR (10 µg/ml) and anti-CD28 (10 µg/ml) respectively for 6 h or 12 h or unstimulated (Unsti-) and then analyzed for CD69 expression.
(1.18 MB TIF)

Figure S2 The expression pattern of transgenic Ckb. (A) Thymocytes from CkbTg or littermate mice were stained for surface CD4, CD8 and intracellular Ckb and then analyzed by flow cytometry. (B) Transgenic expression of Ckb in DP thymocytes is comparable with endogenous Ckb expression in CD4SP thymocytes from littermate controls, shown are two independent experiments (Exp#1 and Exp #2). Litt, littermate; CkbTg, Ckb transgenic.
(1.41 MB TIF)

Figure S3 Detection of surface CD5 and CD69 expression on DP thymocytes. Total thymocytes from TCR transgenic or Ckb and TCR double transgenic mice were stained for surface CD4, CD8 and either CD5 or CD69 and then analyzed by flow cytometry. Litt, littermate; CkbTg, Ckb transgenic.
(1.15 MB TIF)

Figure S4 Flow cytometric analysis of lymph node T cell populations. The percentages of cells within each quadrant are shown in the upper right quadrant. Litt, littermate; CkbTg, Ckb transgenic.
(0.92 MB TIF)

## References

[1] Goldrath AW, Bevan MJ (1999). Selecting and maintaining a diverse T-cell repertoire.. Nature.

[2] Sebzda E, Mariathasan S, Ohteki T, Jones R, Bachmann MF (1999). Selection of the T cell repertoire.. Annu Rev Immunol.

[3] Hogquist KA, Baldwin TA, Jameson SC (2005). Central tolerance: learning self-control in the thymus.. Nat Rev Immunol.

[4] Sprent J, Webb SR (1995). Intrathymic and extrathymic clonal deletion of T cells.. Curr Opin Immunol.

[5] Starr TK, Jameson SC, Hogquist KA (2003). Positive and negative selection of T cells.. Annu Rev Immunol.

[6] Tartaglia M, Mehler EL, Goldberg R, Zampino G, Brunner HG (2001). Mutations in PTPN11, encoding the protein tyrosine phosphatase SHP-2, cause Noonan syndrome.. Nat Genet.

[7] Palmer E (2003). Negative selection - clearing out the bad apples from the T-cell repertoire.. Nat Rev Immunol.

[8] Sprent J, Kishimoto H (2002). The thymus and negative selection.. Immunol Rev.

[9] Jameson SC (2002). Maintaining the norm: T-cell homeostasis.. Nat Rev Immunol.

[10] Mustelin T, Taskén K (2003). Positive and negative regulation of T-cell activation through kinases and phosphatases.. Biochem J.

[11] Qian D, Weiss A (1997). T cell antigen receptor signal transduction.. Curr Opin Cell Biol.

[12] Owen MJ, Venkitaraman AR (1996). Signalling in lymphocyte development.. Curr Opin Immunol.

[13] Rincon M (2001). MAP-kinase signaling pathways in T cells.. Curr Opin Immunol.

[14] Cantrell D (1996). T cell antigen receptor signal transduction pathways.. Annu Rev Immunol.

[15] Feske S (2007). Calcium signalling in lymphocyte activation and disease.. Nat Rev Immunol.

[16] Koretzky GA, Myung PS (2001). Positive and negative regulation of T-cell activation by adaptor proteins.. Nat Rev Immunol.

[17] Hubbard SR, Till JH (2000). Protein tyrosine kinase structure and function.. Annu Rev Biochem.

[18] Cheng AM, Chan AC (1997). Protein tyrosine kinases in thymocyte development.. Curr Opin Immunol.

[19] Knowles JR (1980). Enzyme-catalyzed phosphoryl transfer reactions.. Annu Rev Biochem.

[20] Wyss M, Kaddurah-Daouk R (2000). Creatine and creatinine metabolism.. Physiol Rev.

[21] Ames A (2000). CNS energy metabolism as related to function.. Brain Res Rev.

[22] Tachikawa M, Fukaya M, Terasaki T, Ohtsuki S, Watanabe M (2004). Distinct cellular expressions of creatine synthetic enzyme GAMT and creatine kinases uCK-Mi and CK-B suggest a novel neuron-glial relationship for brain energy homeostasis.. Eur J Neurosci.

[23] Wallimann T, Dolder M, Schlattner U, Eder M, Hornemann T (1998). Creatine kinase: An enzyme with a central role in cellular energy metabolism.. MAGMA.

[24] Wallimann T, Wyss M, Brdiczka D, Nicolay K, Eppenberger HM (1992). Intracellular compartmentation, structure and function of creatine kinase isoenzymes in tissues with high and fluctuating energy demands: the ‘phosphocreatine circuit’ for cellular energy homeostasis.. Biochem J.

[25] Bürklen TS, Schlattner U, Homayouni R, Gough K, Rak M (2006). The creatine kinase/creatine connection to alzheimer's disease: CK-inactivation, APP-CK complexes and focal creatine deposits.. J Biomed Biotechnol.

[26] Balasubramani M, Day BW, Schoen RE, Getzenberg RH (2006). Altered expression and localization of creatine kinase B, heterogeneous nuclear ribonucleoprotein F, and high mobility group box 1 protein in the nuclear matrix associated with colon cancer.. Cancer Res.

[27] Meffert G, Gellerich FN, Margreiter R, Wyss M (2005). Elevated creatine kinase activity in primary hepatocellular carcinoma.. BMC Gastroenterol.

[28] Ishikawa J, Taniguchi T, Takeshita A, Maekawa M (2005). Increased creatine kinase BB activity and CKB mRNA expression in patients with hematologic disorders: Relation to methylation status of the CKB promoter.. Clin Chim Acta.

[29] Huddleston HG, Wong KK, Welch WR, Berkowitz RS, Mok SC (2005). Clinical applications of microarray technology: creatine kinase B is an up-regulated gene in epithelial ovarian cancer and shows promise as a serum marker.. Gynecol Oncol.

[30] Sun G, Liu X, Mercado P, Jenkinson SR, Kypriotou M (2005). The zinc finger protein cKrox directs CD4 lineage differentiation during intrathymic T cell positive selection.. Nat Immunol.

[31] van Deursen J, Heerschap A, Oerlemans F, Rultenbeek W, Jap P (1993). Skeletal muscles of mice deficient in muscle creatine kinase lack burst activity.. Cell.

[32] Sentman CL, Shutter JR, Hockenbery D, Kanagawa O, Korsmeyer SJ (1991). bcl-2 inhibits multiple forms of apoptosis but not negative selection in thymocytes.. Cell.

[33] Amsen D, Calvo CR, Osborne BA, Kruisbeek AM (1999). Costimulatory signals are required for induction of transcription factor Nur77 during negative selection of CD4+CD8+ thymocytes.. Proc Natl Acad Sci U S A.

[34] Bouillet P, Purton JF, Godfrey DI, Zhang L-C, Coultas L (2002). BH3-only Bcl-2 family member Bim is required for apoptosis of autoreactive thymocytes.. Nature.

[35] Punt JA, Osborne BA, Takahama Y, Sharrow SO, Singer A (1994). Negative selection of CD4+CD8+ thymocytes by T cell receptor-induced apoptosis requires a costimulatory signal that can be provided by CD28.. J Exp Med.

[36] Williams LM, Rudensky AY (2007). Maintenance of the Foxp3-dependent developmental program in mature regulatory T cells requires continued expression of Foxp3.. Nat Immunol.

[37] Mahajan VB, Pai KS, Lau A, Cunningham DD (2000). Creatine kinase, an ATP-generating enzyme, is required for thrombin receptor signaling to the cytoskeleton.. Proc Natl Acad Sci U S A.

[38] Chang E-J, Ha J, Oerlemans F, Lee YJ, Lee SW (2008). Brain-type creatine kinase has a crucial role in osteoclast-mediated bone resorption.. Nat Med.

[39] Kuiper JWP, Pluk H, Oerlemans F, van Leeuwen FN, de Lange F (2008). Creatine kinase–mediated ATP supply fuels actin-based evants in phagocytosis.. PLoS Biol.

[40] Mulvaney PT, Stracke ML, Nam SW, Woodhouse E, O'Keefe M (1998). Cyclocreatine inhibits stimulated motility in tumor cells possessing creatine kinase.. Int J Cancer.

[41] Shin J-B, Streijger F, Beynon A, Peters T, Gadzala L (2007). Hair bundles are specialized for ATP delivery via Creatine Kinase.. Neuron.

[42] Debrincat MA, Zhang J-G, Willson TA, Silke J, Connolly LM (2007). Ankyrin repeat and suppressors of cytokine signaling box protein Asb-9 targets Creatine Kinase B for degradation.. J Biol Chem.

[43] Ventura-Clapier R, Kuznetsov A, Veksler V, Boehm E, Anflous K (1998). Functional coupling of creatine kinases in muscles: species and tissue specificity.. Mol Cell Biochem.

[44] Linette GP, Grusby MJ, Hedrick SM, Hansen TH, Glimcher LH (1994). Bcl-2 is upregulated at the CD4+ CD8+ stage during positive selection and promotes thymocyte differentiation at several control Points.. Immunity.

[45] Swainson L, Kinet S, Manel N, Battini J-L, Sitbon M (2005). Glucose transporter 1 expression identifies a population of cycling CD4^+^CD8^+^ human thymocytes with high CXCR4-induced chemotaxis.. Proc Natl Acad Sci U S A.

[46] Yu Q, Erman B, Bhandoola A, Sharrow SO, Singer A (2003). In vitro evidence that cytokine receptor signals are required for differentiation of double positive thymocytes into functionally mature CD8^+^ T cells.. J Exp Med.

[47] Ohashi PS (2003). Negative selection and autoimmunity.. Curr Opin Immunol.

[48] Liu X, Adams A, Wildt KF, Aronow B, Feigenbaum L (2003). Restricting Zap70 expression to CD4+CD8+ thymocytes reveals a T cell receptor-dependent proofreading mechanism controlling the completion of positive selection.. J Exp Med.

[49] Rubinson DA, Dillon CP, Kwiatkowski AV, Sievers C, Yang L (2003). A lentivirus-based system to functionally silence genes in primary mammalian cells, stem cells and transgenic mice by RNA interference.. Nat Genet.

[50] Ioannidis V, Beermann F, Clevers H, Held W (2001). The [beta]-catenin-TCF-1 pathway ensures CD4+CD8+ thymocyte survival.. Nat Immunol.

