# Supplementary figures and images for: Regulation of T Cell Development and Activation by Creatine Kinase B
Source: PLoS One. 2009 Apr 1;4(4):e5000. doi: 10.1371/journal.pone.0005000 (PMC2659424; doi:10.1371/journal.pone.0005000)

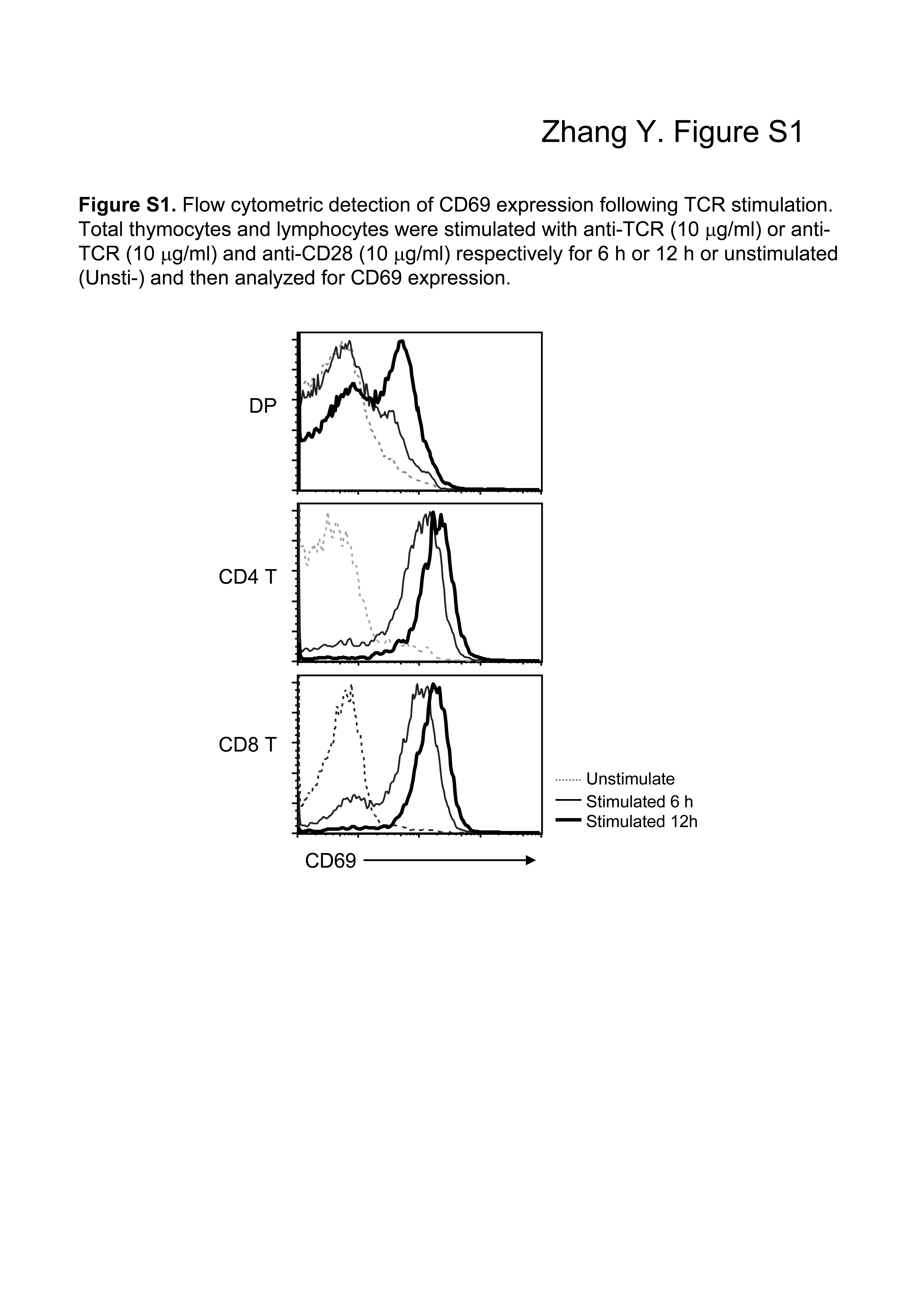

Supplement: Figure S1 — Flow cytometric detection of CD69 expression following TCR stimulation. Total thymocytes and lymphocytes were stimulated with anti-TCR (10 µ/ml) or anti-TCR (10 µg/ml) and anti-CD28 (10 µg/ml) respectively for 6 h or 12 h or unstimulated (Unsti-) and then analyzed for CD69 expression. (1.18 MB TIF) [file pone.0005000.s001.tif]

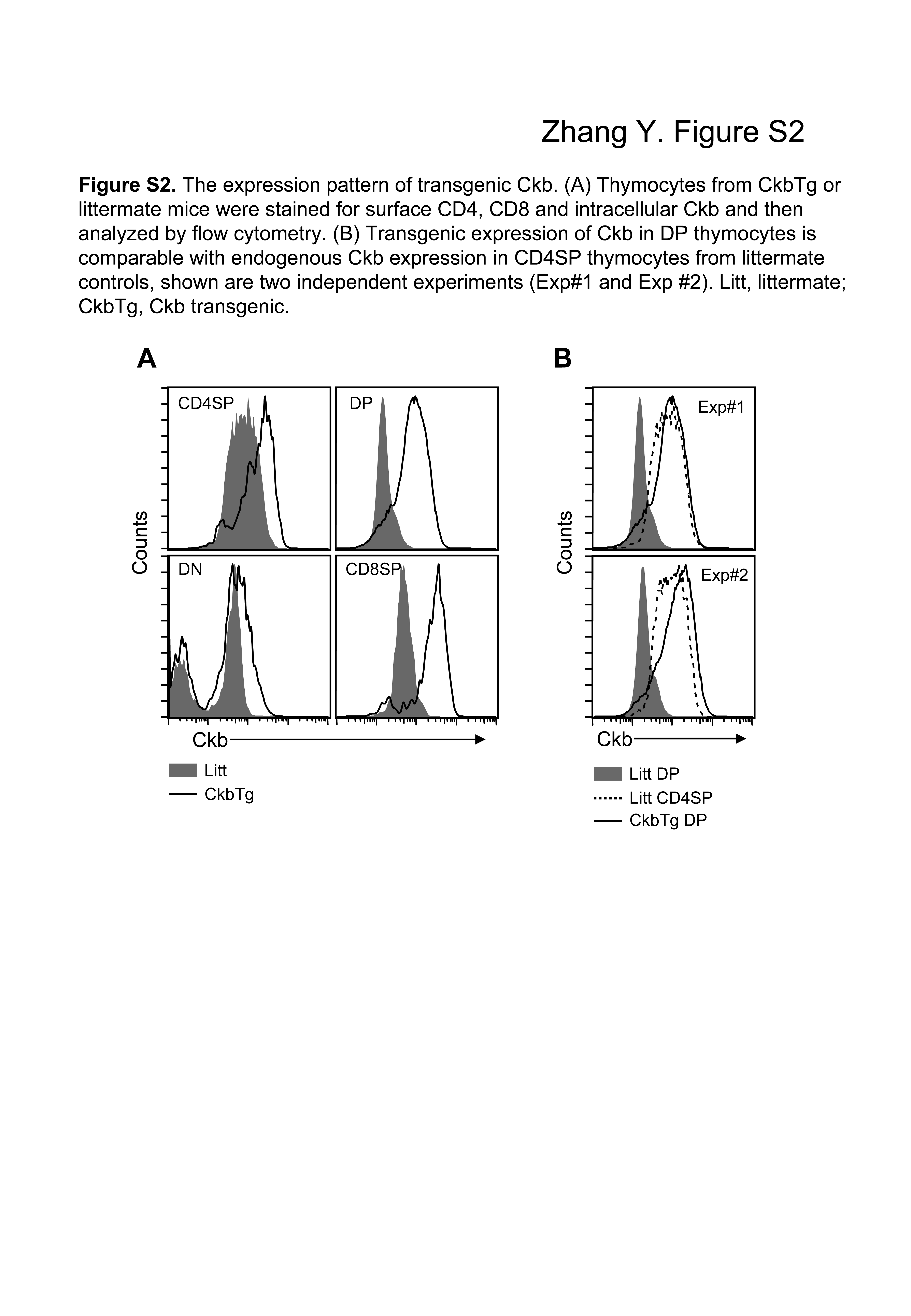

Supplement: Figure S2 — The expression pattern of transgenic Ckb. (A) Thymocytes from CkbTg or littermate mice were stained for surface CD4, CD8 and intracellular Ckb and then analyzed by flow cytometry. (B) Transgenic expression of Ckb in DP thymocytes is comparable with endogenous Ckb expression in CD4SP thymocytes from littermate controls, shown are two independent experiments (Exp#1 and Exp #2). Litt, littermate; CkbTg, Ckb transgenic. (1.41 MB TIF) [file pone.0005000.s002.tif]

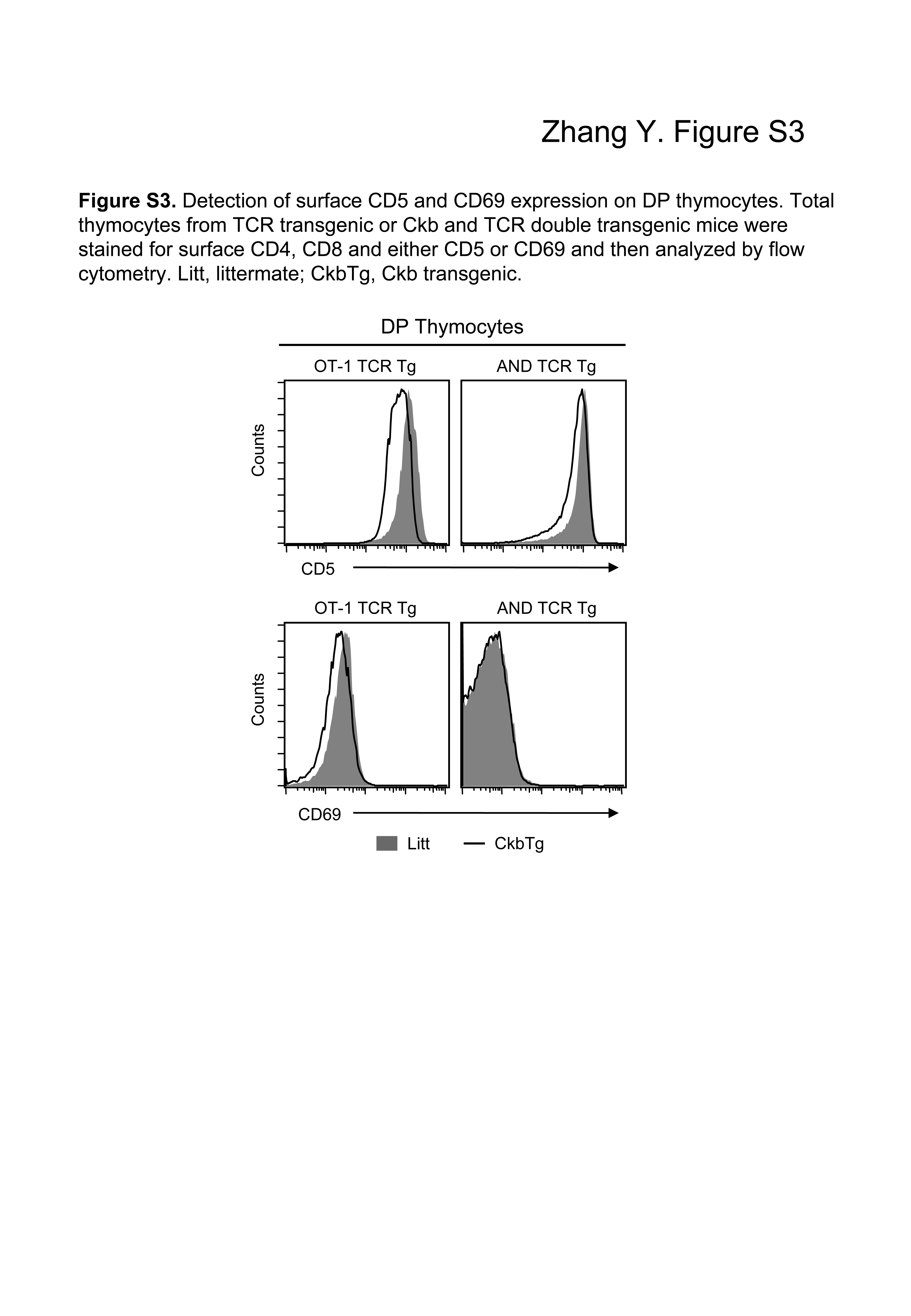

Supplement: Figure S3 — Detection of surface CD5 and CD69 expression on DP thymocytes. Total thymocytes from TCR transgenic or Ckb and TCR double transgenic mice were stained for surface CD4, CD8 and either CD5 or CD69 and then analyzed by flow cytometry. Litt, littermate; CkbTg, Ckb transgenic. (1.15 MB TIF) [file pone.0005000.s003.tif]

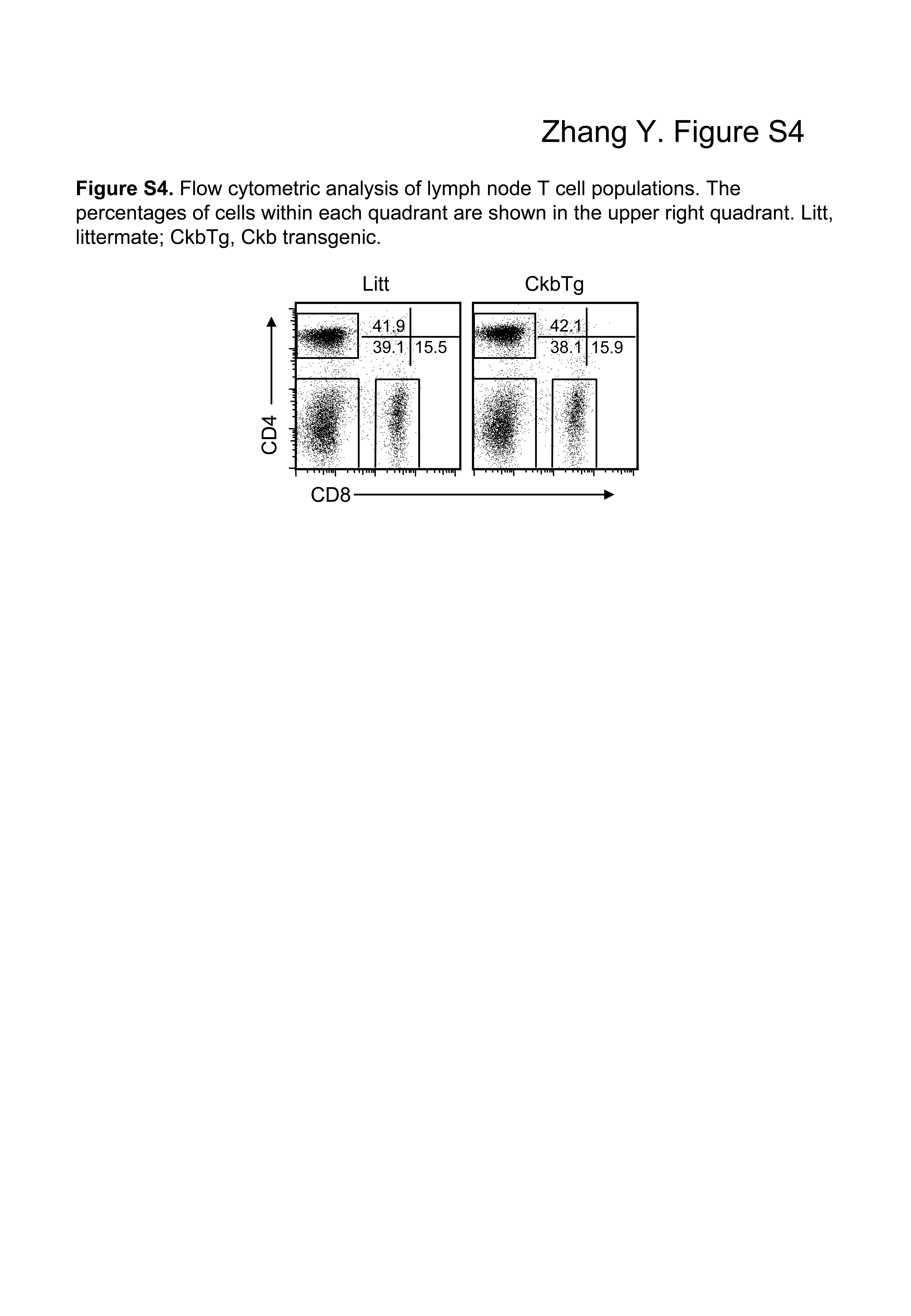

Supplement: Figure S4 — Flow cytometric analysis of lymph node T cell populations. The percentages of cells within each quadrant are shown in the upper right quadrant. Litt, littermate; CkbTg, Ckb transgenic. (0.92 MB TIF) [file pone.0005000.s004.tif]
